# Isolation and characterization of phosphate solubilizing bacteria naturally colonizing legumes rhizosphere in Morocco

**DOI:** 10.3389/fmicb.2022.958300

**Published:** 2022-09-26

**Authors:** Walid Janati, Karima Mikou, Lahsen El Ghadraoui, Faouzi Errachidi

**Affiliations:** Functional Ecology and Environment Engineering Laboratory, Faculty of Science and Technology, Sidi Mohamed Ben Abdellah University, Fez, Morocco

**Keywords:** microbial fertilizers, rock phosphate, stress tolerance, sustainability, antifungal acitivity, TCP

## Abstract

Low-cost and environmentally friendly agricultural practices have received increasing attention in recent years. Developing microbial inoculants containing phosphate (P) solubilizing bacteria (PSB) represents an emerging biological solution to improve rhizosphere P availability. The present study aims to explore PSB strains isolated from soils located at different bioclimatic stages in Morocco and present in various legumes rhizosphere to improve agronomic microbial fertilizer’s effectiveness. It was also aimed to test the isolated strains for their ability to solubilize P in NBRIP medium with Tricalcium P (Ca_3_ (PO_4_)_2_) (TCP), rock phosphate (RP), and their combination as a source of phosphorus, by (2^2^) experiment design. Bacterial strains with a high P solubility index (PSI) were selected, characterized, and compared to commercial control. The vanadate-molybdate method was used to estimate P solubilization activity. Stress tolerance to salinity, acidity, drought, and temperature was tested. From all isolated strains (64), 12 were screened as promising biotechnological interest because of their P solubilization and their good resistance to different drastic conditions. Besides, the strain WJEF15 showed the most P solubility efficiency in NBRIP solid medium with a PSI of 4.1; while the WJEF61 strain was located as the most efficient strain in NBRIP-TCP liquid medium by releasing 147.62 mg.l^–1^ of soluble P. In contrast, in the NBRIP-RP medium, the strain WJEF15 presented maximum solubilization with 25.16 mg.l^–1^. The experiment design showed that a combination of RP and TCP with max level progressively increases P solubilization by 20.58%, while the WJEF63 strain has the most efficient concentration of 102.69 mg.l^–1^. Indeed, among the selected strains, four strains were able to limit tested fungi growth. Thus, results reveal a potential effect of selecting PSBs to support cropping cultures as plant growth-promoting rhizobacteria (PGPR).

## Introduction

Phosphorus (P) is one of the main essential macronutrient compounds for growth and plant development. It is involved in central plant metabolic processes such as cellular energy storage, photosynthesis, and respiration (Balemi and Negisho, 2012; Razaq et al., 2017; Sanz-Sáez et al., 2017; Malhotra et al., 2018). Depending on the environmental and biological factors, it can be the primary growth-limiting nutrient factor (Alok et al., 2013; Rajkumar and Kurinjimalar, 2021). However, the amount of soluble P in soil is generally low (0.4–1.2 g.kg^–1^) (Fernández et al., 2014; Joe et al., 2018). Most parts of soil P (approximately 95–99%) are present in insoluble forms, and therefore they cannot be easily used by plants (Mara et al., 2014). Traditionally, organic and inorganic fertilizers applied to farm fields have been used to correct nutrient deficiencies and maintain nutrient balances (Liu et al., 2020; Tiwari et al., 2020). Soil P is characterized by its restricted mobility when compared to other important nutrients such as nitrogen or potassium and plant nutrient models have long shown that slow diffusion of inorganic phosphate (Pi) is a major limitation to P acquisition by plants (Barber, 1995). This suggests that plants need additional mechanisms to acquire Pi under low P conditions, as their roots have access to a rather small fraction of total soil P. Even if chemical fertilizers are supplied to soils, plants can only use small amounts of P fertilizer because of P complexities in growing weak soil structures. A large quantity of P applied as chemical fertilizer goes out of the plant-soil system through complexity with Ca^2+^ in calcareous soils, and Al^3+^ and Fe^3+^ in acidic soils (Gyaneshwar et al., 2002; Izhar Shafi et al., 2020), therefore, approximately 80% of applied P become unavailable to plant (Salvagiotti et al., 2017). Nevertheless, the negative environmental impact could result from excessive and irrational nutrient application in agroecosystems, which has attracted attention from stakeholders along the food value chain (Choudhury and Kennedy, 2005). According to FAO, more than 175.5 million tons of chemical fertilizers are used in agriculture to obtain the highest crop yield to meet increasing habitat demands.

Most phosphate fertilizers are manufactured using rock phosphate (RP) as the principal source of P_2_O_5_. While demand continues to increase, the supply of rock phosphate is limited worldwide (Suleman et al., 2018). The extremely poor solubility of RP is the only constraint in its direct use as a soil amendment (Baig et al., 2012). However, insoluble P forms such as tricalcium P [Ca_3_ (PO_4_)_2_], aluminum P (AlPO_4_), and iron P (FePO_4_) can be solubilized by organic acids, phosphatase enzymes, and complexing agents produced by soil microorganisms inhabiting different soil ecosystems (Vaishampayan et al., 2001). Several studies have reported the usage of microorganisms to solubilize insoluble phosphate compounds as an alternative strategy to phosphate fertilizers (Adnan et al., 2020). Indeed, using these telluric competent strains is an agroecological strategy for improving agricultural fertility and reducing farmers’ dependency on inorganic fertilizers to enhance soil health (Janati et al., 2021). For several decades, agricultural microbiologists have studied the ability of certain bacteria to dissolve P fertilizers and P chelated in soil according to sustainability tendency. For this purpose, Behera et al. (2017) noted that PSB’s ability to solubilize P from soil or fertilization activities in many agricultural fields is highly dependent on their secretion of organic acids such as citric, formic, oxalic, lactic, acetic, and malic acids. Moreover, PSBs can increase plant P availability by modifying soil processes in the rhizosphere, providing essential nutrients to plants and generating other regulators’ growth in various production systems (Adnan et al., 2020; Islam et al., 2021). Likewise, PSBs can produce growth-promoting hormones such as Gibberellins and Indol Acetic Acid (IAA), which are beneficial for plant growth (Bahadur et al., 2016; Linu et al., 2019; Khan et al., 2020; Kour et al., 2020). Currently, most bacteria classified as PSBs belong to *Pseudomonas*, *Serratia*, *Burkholderia*, *Achromobacter*, *Agrobacterium*, *bacillus*, *rhizobia*, *Micrococcus*, *Aerobacter*, *Flavobacterium*, *Acinetobacter*, and *Pantoea* genera (Castagno et al., 2011; Chauhan A. et al., 2015; Adnan et al., 2019; Seenivasagan and Babalola, 2021).

Morocco covers a significant share of world P reserves; it contains 75% of planetary P and is the leading exporter of P and its derivatives. So the global market country’s contribution is more than 30% (Hakkou et al., 2016). This advantageous position of Morocco on commercial and technical levels of P extraction remains very far from a bio-industry that accompanies the sector of biotechnology. The latter could open up possibilities for the maximum valorization of soil P. Although PSBs can provide agronomic benefits, they are not always abundant enough in the soil to compete with other indigenous microorganisms (Acevedo et al., 2014). For this reason, these telluric bacteria should be promising at the same time crop productivity and economic-environmental sustainability need to be selected, characterized, and identified to establish sustainable cropping systems.

Considering the above facts, the present study focused on the effect of several tolerant PSBs on different abiotic stresses to promote P solubilization from RP and TCP as the only P source in the context of biological fertilization and sustainable agricultural strategy. We isolated and characterized PSBs from legumes rhizosphere located at various bioclimatic stages and we detected their optimal resistance under different growth and environmental stress conditions (salinity, pH, drought, and temperature). The findings of this study could provide an effective approach for agronomic improvement of microbial inoculants to enhance soil P mobilization for plant growth, either in intensive production or in agroecological applications in Morocco.

## Materials and methods

### Soil sample collection and analysis

Soil samples were taken from different rhizospheres of cultivated legumes, cereals, and spontaneous plants from different localities in Fes-Meknes regions. Plantations are located at different bioclimatic stages in four stations, namely Tafrant, Ait Saleh, Dayt Al Amira, and Ait Yaakoub. These locations are characterized by:

• Tafrant: Climate is warm and temperate, with less rainfall in winter. The average annual temperature is 18.1^°^C and average annual rainfall is 866 mm.
• Ait Saleh: Climate is warm and temperate. Rain falls mainly in winter, with relatively little rain in summer. It has an average annual temperature of 15.5^°^C and an average annual rainfall of 651 mm.
• Dayt Al Amira: Climate is warm and temperate. Rainfall is high even in the driest months. The average annual temperature is 12.1^°^C and the average annual rainfall is 683 mm.
• Ait Yaakoub: Climate is warm and temperate. In winter, rainfall is much higher than in summer. The average annual temperature is 17.8^°^C and the average annual rainfall is 549 mm (Climate-data.org, 2022).

Rhizospheric soil samples were carefully collected, placed in labeled sterile plastic bags, analyzed for physico-chemical characteristics and stored at –20^°^C before rhizobacteria isolation.

### Isolation and purification of phosphate solubilizing bacteria

A soil suspension was prepared to isolate PSBs from a dilution cascade. For this purpose, 10 g of soil were suspended in 100 mL of sterile phosphate-buffered saline (PBS) solution (pH 7.2) and shaken at 190 rpm for 45 min. Subsequently, serial dilutions were prepared to 10^–6^. About 100 μL of each dilution was placed on NBRIP agar plates (National Botanical Research in P medium), which has as composition: glucose 10 g, (NH_4_)_2_SO_4_ 0.1 g, MgSO_4_.7H_2_O 0.25 g, KCl 0.2 g, and agar 15 g, supplemented with 5 g RP from Khouribga phosphate mine and dissolved in 1,000 ml distilled water, and the medium is stabilized to pH 6.8 (Nautiyal, 1999). The NBRIP medium was then complemented with cycloheximide to inhibit fungal growth. NBRIP agars plates were incubated for 6 days at 28 ± 2^°^C. During this incubation, clear zones of solubilization surrounding the colony allowed the detection of PSBs. Every different morphological colony types were isolated, purified, and retested by restricting them from a single colony (five times) for purification on NBRIP plates and finally preserved in Tryptic Soy Agar (TSA) medium before cryopreservation in sterile glycerine (20%) at –80^°^C.

### Quantitative estimation of phosphate solubilization indice

Colony diameter and visible halos were evaluated between 3 and 9 days after inoculation to calculate the P solubilization index (PSI) (Nautiyal, 1999). Halo and colony diameters were measured after 9 days of incubation on NBRIP plates’ at 28 ± 2^°^C. Bacteria’s ability to solubilize insoluble P was described by the solubilization index formula (Equation 1):


(1) $$\text{PSI}=\frac{C\,D+H\,D}{C\,D}$$

**CD:** Colony Diameter.

**HD:** Halo zone Diameter.

### Quantitative estimation of phosphate solubilization using tricalcium phosphate, rock phosphate, and their combination

#### Phosphate solubilizing assessment

Phosphate solubilizing bacteria’s quantitative solubilization capacity of TCP, RP, and their combination was done in a liquid NBRIP medium. For this purpose, overnight pre-culture in TSA medium was prepared and then moved to NBRIP medium. After 7 days of incubation at 28°C under shaking to 150 rpm, 20 ml of bacterial suspensions were taken and centrifuged (12,000 g for 15 min). Supernatants were recovered and analyzed for their assimilable P content and pH measurements. Assimilable P content was carried out by the ascorbic acid colorimetric method (Murphy and Riley, 1962). About 1 mL of supernatant is mixed with 160 μL of a reaction solution and the OD (optical density) was then measured at 880 nm after a few minutes of incubation at room temperature. Phosphate soluble content was calculated based on a standard P solution of KH_2_PO_4_ (concentrations ranging from 0 to 1 mg.l^–1^).

#### Optimization study of combined rock phosphate and tricalcium phosphate solubilization system

Optimization strategy to reveal and quantify the influence of PSBs effect on the solubilization process of two combined phosphate sources (RP and TCP) was conducted following a two-level experimental design (2^2^ = 4 experiments). Experimental domain of both factors [X_1_ = (RP) and X_2_ = (TCP)] are presented in Table 1. The experimental design was developed to study the effects of the two factors (X_1_ and X_2_) and their interaction (X_1_*X_2_) on the concentration of P solubilized and pH medium (Table 2).

**TABLE 1:** Factors’ levels (X1 and X2) involved in P solubilization.

	Min level	Max level
	–1	+1
X_1_ : RP	1%	2.5%
X_2_ : TCP	1%	2.5%

**TABLE 2:** Experimental design developed to study the effects of factors (X1: RP and X2: TCP) and their interaction (X1*X2) on pH medium and P concentration.

Experiment	X_0_	X_1_	X_2_	X_1*2_	Y_P[C]_	Y_pH_
1	+1	–1	–1	+1	–	–
2	+1	–1	+1	–1	–	–
3	+1	+1	–1	–1	–	–
4	+1	+1	+1	+1	–	–

Y_P[C]_: concentration of P solubilized response function. Y_pH_: pH medium response function.

#### Factors and their mathematical modeling interaction

The matrix analysis model of experimental design (Table 2) results in response functions indicating the coded factors’ effects (X1 and X2) and their interaction, which has the following formula (Equation 2):


(2) Y=a+0aX1+1aX2+2aX12X12

**a_0_:** Coefficient model.

**a_1_** and **a_2_:** Coefficients of the linear part of the mathematical model.

**a_12_:** Interaction coefficient of the two factors.

Mathematical analysis interactions between factors (X_1_ and X_2_) provide response surfaces and allow us to define the domains where there is a maximum or minimum response of the studied response functions (Y_*P[C]*_ and Y_*pH*_) experimentally.

#### Morphological and biochemical characterization

Phosphate solubilizing bacteria were characterized as described in Bergey’s Manual (Systematic Bacteriology, 2nd ed., vol. 5, eds., 2012), and cells morphology, motility test, and Gram staining were performed (Aneja, 2007). Isolates were also tested for catalase, gelatinase, and urease activities (Graham and Parker, 1964; Aneja and Wilkes, 2003). Experiments were repeated three times for each isolate. To determine PSBs’ capability to use starch as a source of carbon (Salgaonkar et al., 2018), PSBs were inoculated on starch agar media and incubated at 28^°^C ± 2^°^C for 24 h. Citrate utilization by the isolates was observed by growth on YEM agar containing sodium citrate in place of mannitol and supplemented with 1 ml of bromothymol blue (BTB) solution (1%) and incubated at 30°C. After incubation (7 days), a distinct change in color, from green to blue, refers to as a positive test. A glucose peptone agar (GPA) assay was performed to determine PSB’s capability to use glucose as the sole source of carbon and energy for growth (Amela et al., 2018). Similarly, a lactose assay was performed (Paudyal et al., 2021).

#### Bacterial stress tolerance

Isolates salinity tolerance evaluation was done onto a liquid rich medium (TSA) containing different NaCl concentrations (0.2, 0.4, 0.6, 0.8, 1, 1.2, 1.6, and 2 M). Incubation was carried out at 28°C ± 2°C for 48 h. Then, absorbance was measured at 620 nm (Singh et al., 2015). As solubilization is strongly related to pH medium changes, isolates were inoculated on a solid TSA medium at different pH values starting from 3 to 13 and incubated at 28°C ± 2°C for 48 h. This test allows isolates selection that can grow over a wide range of soil pH (acid, neutral, and alkaline). Phosphate solubilizing bacteria isolates were also cultured onto a solid rich agar medium (TSA) and incubated at 37, 48, and 55°C for 48 h to assess isolates’ temperature tolerance through bacterial growth. In addition, drought tolerance of PSBs was assessed on a liquid medium (TSA) containing different concentrations of polyethylene glycol 6000 (PEG) (10, 20, and 30%). Incubation was carried out at 28°C ± 2°C for 24 h, after that absorbance assessment was done at 620 nm.

#### Antifungal effect of phosphate solubilizing bacteria strains

All 12 bacterial isolates were tested for their antagonistic effect against six fungal strains isolated from strawberry fruit and grown in a PDA medium. To assess antagonism, a 5-mm block (growing mycelium + medium) was placed in the middle of the Petri dish. Tested bacteria were placed in four different positions around the block. After incubation at 26°C ± 2°C for 24 h, a zone of inhibition between colonies and fungal strains indicates antagonism presence. Finally, isolates producing more than 15 mm of inhibition zone were selected. The inhibition rate of fungal growth was determined using the percentage of inhibited radial growth (% IRG) according to the following formula (Equation 3):


(3) $$\%\text{IRG}=\frac{\text{R1}-\text{R2}}{\text{R1}}*100$$

**R_1_** radial growth of control mycelium.

**R_2_** radial growth of treated mycelium.

### Statistical analysis

Data were analyzed using SPSS software, and the results were expressed as the means ± standard deviation of three replicates. Data were examined by ANOVA I analysis, and mean comparison was performed by Duncan’s multiple range test at *p* ≤ 0.05. Dimensional analysis done by principal component analysis (PCA) has been used to give a summarizing view of obtained results. For the design of the experiment trial, all experiments were done in four replicates, and data were analyzed using JMP 4 software. Response surface of experimental design response functions (Y_*pH*_ and Y_*P[C]*_) were done by Maple 9 software.

## Results

### Soil physico-chemical analysis

Soil samples’ physico-chemical and PSBs contents are listed in Table 3. Agricultural legume soils analyzed contained, on average, 4.4% of Organic Matter, 7.9 (mg/Kg) of nitrogen, 44.65 (mg/Kg) of P_2_O_5_, 660.5 of K_2_O (mg/Kg), 7,406.25 of CaO (mg/Kg), and 848.83 (mg/Kg) of MgO. Maximum electrical conductivity, reflecting soil salinity, was recorded in the Ait Yaakoub soil sample with an average of 300.33 (ds/m). In terms of soil pH, it was close to neutral or slightly basic (pH = 8.2 on average).

**TABLE 3:** Physico-chemical and PSBs analysis of the studied soil samples.

Sites	Tafrant	A. Yaakoub	D.A. Amira	A. Saleh
Coordinates	**Y** –5.112751, **X** 34.615013	**Y** –4.950365, **X** 33.975209	**Y** –5.019709, **X** 33.692496	**Y** –4.992072, **X** 33.791152
Altitude (m)	312.5	566	1548	922
T.avg/year (^°^C)	18.1	17.8	12.1	15.5
P.avg/year (mm)	866	549	683	651
PSBs load (Cells/g of soil)	5.95*10^3^	1.98*10^5^	1.04*10^6^	3.16*10^7^
pH-water	8.26	8.323	8.067	8.273
Electrical conductivity 1/5 (ds/m)	187.033	300.333	241.233	173.2
Cation exchange capacity (cmol+/kg)	40.987	32.28	37.227	31.563
Organic matter (%)	2.573	5.103	5.64	4.38
N total (%)	0.126	0.255	0.324	0.174
N-NH_4_ + N-NO_3_ (mg/kg N)	7.383	8.867	8.167	7.4
P_2_O_5_ (mg/kg)	10	46.7	98.7	23.3
K_2_O (mg/kg)	432	846.3	990.7	373
CaO (mg/kg)	9,450	8,550	5,250	6,375
MgO (mg/kg)	410.667	854	1213.333	917.333

**FIGURE 1:**
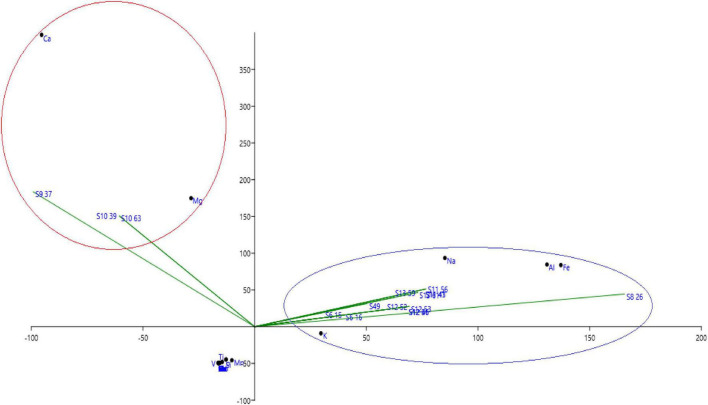
Principal component analysis of different soil in the function of mineralogical composition.

The mineralogical compositions of different analyzed sites with ICP are presented, respectively, in Figure 1. We notice that Al, Ca, Fe, K, Mg, Na, and P are the most important minerals in the studied sites. Based on principal component analysis of different sites in terms of mineralogical composition, we find that sites S9 (Dayt Al Amira) and S10 (Ait Saleh) are controlled by both Mg and Ca minerals, while all the other sites are controlled by Al, Fe, K, and Na minerals.

### Selection and purification of phosphate solubilizing bacteria

Soil samples collected from different sites were used to study the PSB bacteria. Strains were selected based on colonies’ morphological difference and halo resulting from TCP solubilization. Although, halo formation around colonies provides the first qualitative indication of PSB. Colonies that grew without forming a halo were also isolated to test their solubilization capacity. Initially, 134 isolates were selected; these isolates were transplanted onto the NBRIP medium three times to guarantee their efficiency and stability. Among 134 isolates, 64 were designated as PSB based on their solubilization of P on NBRIP liquid medium, and only 12 isolates were screened as promising biotechnological interest.

### Quantitative estimation of phosphate solubilization indice

**FIGURE 2:**
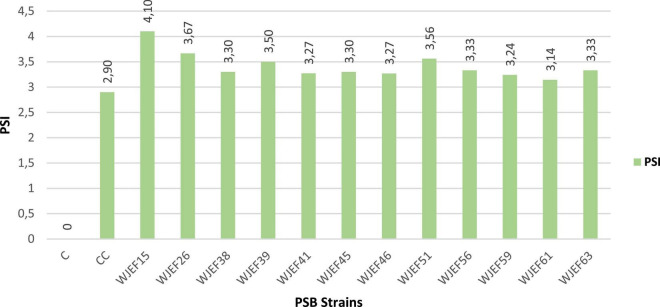
Phosphate solubilization index (PSI) of isolated strains on solid NBRIP medium.

The appearance of the halo zone in all strains was noticed after 4th day of incubation, solubilization index ranged from 3.14 cm to 4.1 on average. Isolate WJEF15 showed a maximum solubilization index with PSI = 4.1 (Figure 2). Thus, a significant difference was found for all strains tested. We notice the existence of a correlation between incubation time and halo zone size, whereby the halo zone size of each isolate increases with incubation time.

### Quantitative estimation of phosphate solubilization using tricalcium phosphate and rock phosphate

**FIGURE 3:**
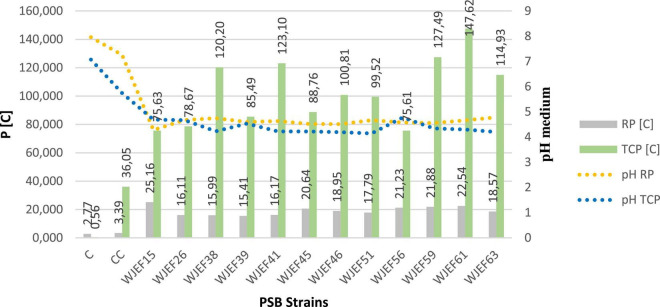
PSBs solubilization capacity of TCP and RP after 7 days in liquid medium.

Soluble P and pH variations are presented in Figure 3. The solubilization of TCP, RP, and their combination in liquid NBRIP medium by tested strains was accompanied by a significant pH decrease. NBRIP-TCP soluble P concentration varied between 75.61 and 147.62 mg.l^–1^, whereas NBRIP-RP soluble P concentration varied between 15.41 and 25.16 mg.l^–1^ with significant variations between all strains when compared to control (*p* ≤ 0.05). However, maximum solubilization in NBRIP-TCP liquid medium was observed for isolate WJEF61 (147.62 mg.l^–1^). In contrast, for the NBRIP-RP medium, strain WJEF15 presented maximum solubilization with 25.16 mg.l^–1^. On the other hand, commercial control solubilized only 36.05 mg.l^–1^ of P with TCP and 3.39 mg.l^–1^ with RP. Besides, NBRIP medium pH inoculated with selected strains strongly decreased from 7.06 to 4.145 (TCP) and from 7.97 to 4.28 (RP) after 7 days of incubation. Compared to the commercial control, it decreased to 5.74 and 7.26, respectively.

### Correlation between pH medium and solubilization concentration

**FIGURE 4:**
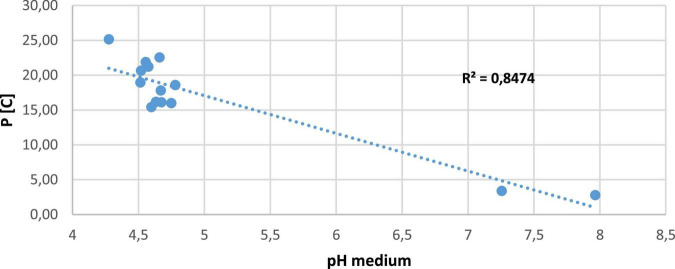
Negative correlation between pH medium and solubilization concentration.

The correlation between P[C] and pH was positive in all strains with high P solubility. Correlation coefficients (R) were greater than 0.84 for P [C] vs. pH medium. However, in several applications, regression equations for P [C] vs. pH medium were linear models (Figure 4).

### Design of experiments method to study tricalcium phosphate and rock phosphate interaction

Experimental matrix analysis design (Tables 4, 5) results (response functions) present coded factors’ effects (X_1_ and X_2_) and their interaction (X_1_*X_2_) (Equation 2). The control (non-inoculated) was introduced to evaluate the chemical reaction of the medium.

**TABLE 4:** Effect of phosphorus source (RP and TCP) on culture medium acidification.

Isolates	Model coefficient	RP effect	TCP effect	Interaction RP/TCP
Coefficients	a_0_	a_1_	a_2_	a_12_
Control	1.931	0.013	0.055	–0.016
Commercial control	0.335	–0.025	–0.079	0.016
WJEF15	3.278	–0.034	0.029	0.047
WJEF56	2.793	0.042	–0.019	–0.101
WJEF26	2.921	–0.020	–0.138	–0.019
WJEF38	3.237	0.063	0.047	–0.129
WJEF41	3.193	0.020	0.075	–0.143
WJEF45	3.222	0.011	0.146	–0.123
WJEF46	3.209	0.057	0.104	–0.076
WJEF51	3.261	0.009	0.101	–0.149
WJEF59	3.089	0.084	0.100	–0.115
WJEF61	3.237	0.051	0.092	–0.109
WJEF63	3.199	0.085	0.140	–0.161

**TABLE 5:** Impact of phosphorus source (RP and TCP) on P solubilization in culture medium.

Isolates	Model coefficient	RP effect	TCP effect	Interaction RP/TCP
Coefficients	a_0_	a_1_	a_2_	a_12_
Control	0.481	–0.055	–0.031	–0.049
Commercial control	1.076	–0.030	0.178	–0.037
WJEF15	14.637	–0.598	8.050	–0.405
WJEF56	10.102	0.228	4.623	–1.197
WJEF26	10.865	–2.116	3.722	–0.898
WJEF38	15.491	–1.406	7.430	–0.873
WJEF41	14.208	0.570	8.394	–2.034
WJEF45	14.513	0.912	8.628	–1.993
WJEF46	14.738	0.966	8.768	–1.774
WJEF51	16.397	–0.182	8.601	–1.119
WJEF59	16.196	0.008	7.785	–1.589
WJEF61	16.802	0.713	8.019	–1.779
WJEF63	16.574	1.452	8.743	–1.094

Phosphate solubilization and pH medium results obtained by WJEF26 isolate according to the experimental design are summarized in Table 6. The values correspond to the mean of four replicates.

**TABLE 6:** Experimental design results done after P solubilization by isolate WJEF26.

Experiments	X_1_	X_2_	X_12_	Y_pH_	Y_P[C]_
1	–1	–1	+1	4.3	33.442
2	+1	–1	–1	4.7725	23.696
3	–1	+1	–1	5.1175	70.405
4	+1	+1	+1	5.165	46.289

Y_pH_: Function response of pH medium during P solubilization. Y_P[C]_: Function response of P solubilization (mg.l^–1^).

**FIGURE 5:**
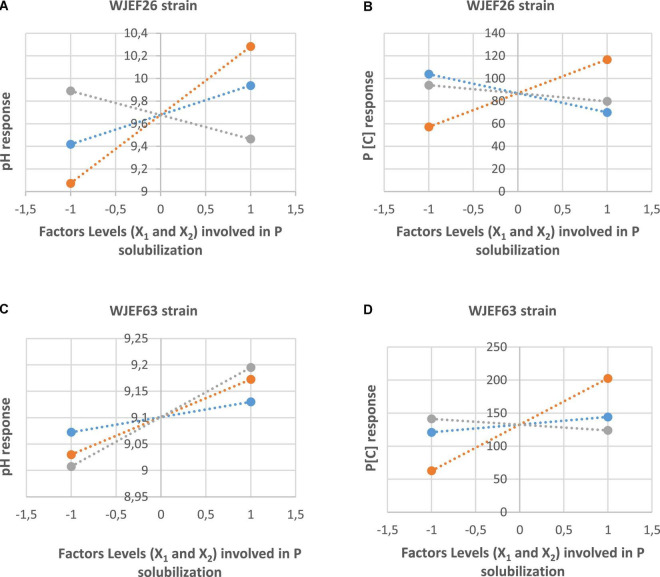
**(A)** Effects of studied factors (X_1_ and X_2_) and their interaction on pH medium in the presence of WJEF26 strain. **(B)** Effects of studied factors (X_1_ and X_2_) and their interaction on P[C] in the presence of WJEF26 strain. **(C)** Effects of studied factors (X_1_ and X_2_) and their interaction on pH medium in the presence of WJEF63 strain. **(D)** Effects of studied factors (X_1_ and X_2_) and their interaction on P[C] in the presence of WJEF63 strain.

The main factors’ effects (X_1_ and X_2_) and their interaction (X_1_*X_2_) of the WJEF26 strain, for example, are summarized in Figures 5A,B. We notice that factors’ interaction on pH medium and P [C] have a positive effect in comparison to each factor alone.

For the strain WJEF63, P solubilization and pH medium results obtained according to the designed experiment are summarized in Table 7. Values correspond to the four replicates’ mean.

**TABLE 7:** Experimental design results done after P solubilization by isolate WJEF63.

Experiments	X_1_	X_2_	X_12_	Y_pH_	Y_P[C]_
1	–1	–1	+1	4.547	21.144
2	+1	–1	–1	4.482	41.505
3	–1	+1	–1	4.525	99.836
4	+1	+1	+1	4.647	102.699

Y_pH_: Function response of pH medium during P solubilization. Y_P[C]_: Function response of P solubilization (mg.l^–1^).

Factors’ effects (X_1_ and X_2_) and their interaction (X_1_*X_2_) of WJEF63 isolate are summarized in Figures 5C,D. We notice that factors’ interaction on P [C] has a positive effect on the contract of each factor alone. On the other hand, the effect of factors alone or in interaction on pH medium have the same activity.

**FIGURE 6:**
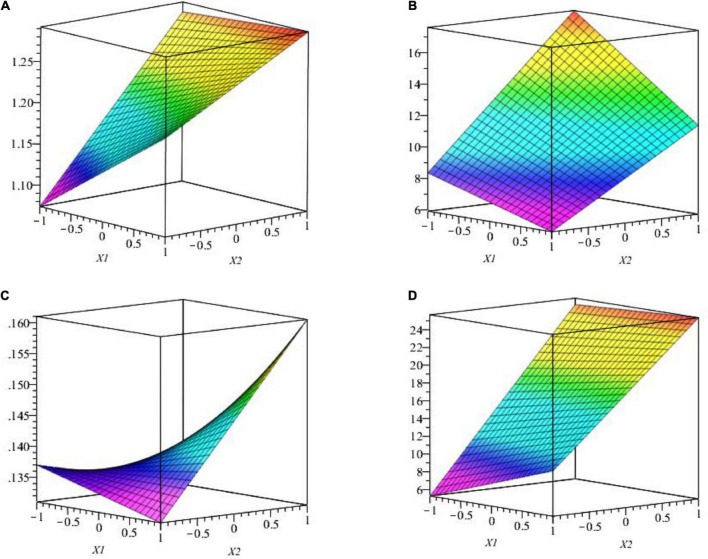
**(A)** The effect of studied factors (X_1_ and X_2_) and their interaction on pH surface response by WJEF26 strain. **(B)** The effect of studied factors (X_1_ and X_2_) and their interaction on the response surface of P[C] by WJEF26 strain. **(C)** Effect of studied factors (X_1_ and X_2_) and their interaction on response surface of pH with WJEF63 strain. **(D)** Effect of studied factors (X_1_ and X_2_) and their interaction on response surface of P[C] with WJEF63 strain.

Factor interaction is presented in Figure 6, where we observe the response surface according to coded variables (RP medium and TCP) which delimits the experimental domain. Figure 6A shows that the pH medium is maximal when RP is at the lowest level and TCP is at the highest level (1 and 2.5%, respectively). On the opposite, Figure 6B shows that P[C] is maximal when both factors X_1_ and X_2_ are at the highest level (2.5 and 2.5%, respectively).

The second example was done with the WJEF63 strain. The interaction of studied factors is presented in Figures 6C,D, where we observe the response surface according to coded variables (RP medium and TCP) which delimits the experimental domain. The figure shows that pH medium and P [C] is maximal when RP is at the lowest level and TCP is at the highest level (1 and 2.5%, respectively). From response surface function, we notice that strains’ metabolic response depends on the nature of the used microorganisms.

### Morphological and biochemical characteristics

The morphological profile of studied strains shows a whole appearance; irregular edges, white, off-white, or yellow color, and a mucoid or viscous texture. Cell structures show rod and coccobacillus shapes with an arrangement of single pairs, chain rods, and solitaires. Gram reaction indicates that all isolates were Gram-negative with the exception of five strains (WJEF15, WJEF38, WJEF41, WJEF45, and WJEF59). Cell motility test and catalase activity is positive for all selected strains (Table 8). Similarly, all strains grew on GPA with glucose or lactose as a source of carbon. The same results were obtained from the starch hydrolysis test except for WJEF61, WJEF52, and WJEF56 strains. After submitting inoculated plates to iodine vapor, clear areas around colonies were observed and the colonies turned yellow, while blue color occurs on growth-free areas. This indicates that two strains (WJEF56 and WJEF61) have the potential to hydrolyze starch present in the medium.

**TABLE 8:** Morphological and biochemical characteristics of selected strains.

PSB strains/characteristics		Commercial control	WJEF15	WJEF26	WJEF38	WJEF39	WJEF41	WJEF45	WJEF46	WJEF51	WJEF56	WJEF59	WJEF61	WJEF63
Colony morphology	Margin	I	E	E	E	E	I	E	I	E	I	I	E	E
	Color	W	Y	Y	OW	Y	OW	OW	OW	OW	Y	Y	OW	OW
	Texture	D	V	V	V	V	M	V	M	V	M	M	V	V
Cells morphology	Shape	R	R	R	C	R	C	C	C	C	C	C	C	CB
	Arrangement	S	S	CR	S	S	S	S	S	SP	S	S	S	S
Physiological tests	Gram staining	+	+	–	+	–	+	+	–	–	–	+	–	–
	Catalase	+	+	+	+	+	+	+	+	+	+	+	+	+
	Motility	+	+	+	+	+	+	+	+	+	+	+	+	+
	Gelatin hydrolysis	–	+	+	–	+	–	–	–	–	+	+	–	–
	Urea hydrolysis	–	–	–	+	–	–	–	–	–	–	–	–	–
	GPA (Glucose)	+	+	+	+	+	+	+	+	+	+	+	+	+
	GPA (Lactose)	+	+	+	+	+	+	+	+	+	+	+	+	+
	Starch	–	–	–	–	–	–	–	–	–	+	–	+	–

Tested positive, utilized substrate; tested negative, not utilized substrate. E, entire; I, irregular; Y, yellow; W, white; OW, off-white; D, Dry; V, viscid; M, mucoid; R, rod; C, cocci; CB, coccobacillus; SP, single pairs; CR, chain rods; and S, solitaire.

### Bacterial tolerance to stresses

According to the results in Table 9, it is evident that selected strains are tolerant to considerable variations in terms of alkalinity, acidity, salinity, dryness, and temperature. All strains present tolerate temperatures between 24 and 37^°^C, except the strain WJEF56. Also, strains WJEF45, WJEF46, WJEF51, WJEF61, and WJEF63 were able to grow at temperatures up to 55°C. In addition, these isolates were able to grow and survive over a wide pH range (3–13). Moreover, among all tested isolates, only WJEF45, WJEF51, WJEF59, and WJEF61 were able to successfully resist higher NaCl concentrations (2M) g/L (Table 9).

**TABLE 9:** Physiological characteristics of selected isolates under temperature, pH, and salinity pressure.

PSB strains/pressures		Commercial control	WJEF15	WJEF26	WJEF38	WJEF39	WJEF41	WJEF45	WJEF46	WJEF51	WJEF56	WJEF59	WJEF61	WJEF63
GT	24°C	1	1	1	1	1	1	1	1	1	1	1	1	1
	37°C	1	1	1	1	1	1	1	1	1	–1	1	1	1
	48°C	1	–1	–1	1	1	1	1	1	1	1	1	1	1
	55°C	–1	–1	–1	–1	–1	–1	1	1	1	–1	–1	1	1
G pH	pH 3	–1	1	1	1	1	1	1	1	1	1	1	1	1
	pH 5	1	1	1	1	1	1	1	1	1	1	1	1	1
	pH 7	1	1	1	1	1	1	1	1	1	1	1	1	1
	pH 9	1	1	1	1	1	1	1	1	1	1	1	1	1
	pH 11	1	1	1	1	1	1	1	1	1	1	1	1	1
	pH 13	–1	1	1	1	1	1	1	1	1	1	1	1	1
GS	1.6M	1	1	1	–1	1	1	1	1	1	–1	1	1	1
	2M	–1	–1	–1	–1	–1	–1	1	–1	1	–1	1	1	–1

GT, growth temperature; GpH, growth pH; GS, growth salinity. Tested positive/utilized as substrate; tested negative/not utilized substrate.

**FIGURE 7:**
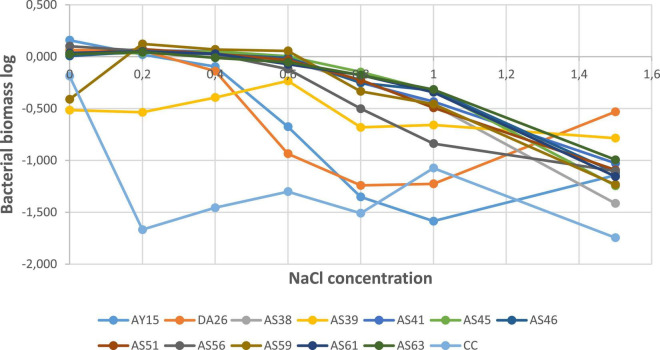
Biomass in function of salt concentrations g/L.

To quantify tested PSBs’ tolerance to high salt concentrations, we incubated PSBs in a nutrient-liquid medium, and after 24 h, we measured biomass formation expressed by absorbance. Log (AB) as a function of salt concentrations revealed that all strains significantly decreased at 1.5M NaCl concentration, except WJEF15, WJEF26, and WJEF39 strains, which resist and show positive growth (Figure 7).

**FIGURE 8:**
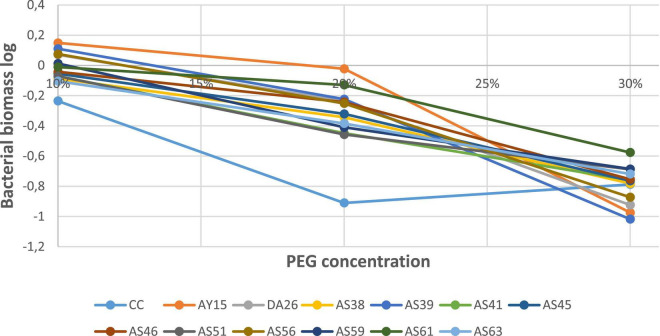
Biomass in the function of PEG concentrations.

Regarding drought tolerance, all 12 isolates show the same behavior under increasing PEG-6000 concentrations (10, 20, and 30%). PSB isolates exhibit a decline in cell numbers and absorbance value. However, the WJEF61 strain presented an optimal resistance, with an absorbance value higher than those of the other isolates and commercial control to all PEG levels (Figure 8).

**FIGURE 9:**
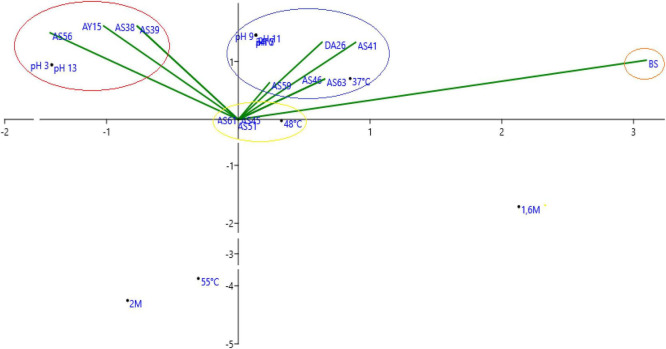
Principal correspondence analysis of tested isolates on physico-chemical pressure.

Based on the principal correspondence analysis of tested strains on physico-chemical pressure, we notice that we have two major groups of strains, the first one is correlated with temperature and the second group is correlated with alkalinity, acidity, and salinity (Figure 9).

### Antifungal effect of phosphate solubilizing bacteria strains

Fungal growth inhibitory of isolated PSBs was tested on six plant pathogenic fungi: *Aspergillus Niger, Fusarium Culmorum, Fusarium Oxysporum, Penicillium Digitatum, Penicillium Notatum*, and *Rhizopus Stolonifer* (Table 10). The antifungal activity showed that of the 12 bacteria, 3 (WJEF26, WJEF56, and WJEF61) were able to inhibit fungal growth of at least 4 fungi tested (Table 10). In addition, the best antagonist against tested fungi was located in the WJEF61 strain with an IRG percentage of 49.15, 23.51, 32.76, and 32.92% against *Aspergillus Niger*, *Fusarium Culmorum*, *Fusarium Oxysporum*, and *Rhizopus Stolonifer*, respectively. The WJEF59 strain has an antagonistic effect only against *R. Stolonifer* with a % IRG of 36.54% which presents a specific character (Table 10).

**TABLE 10:** Fungal inhibited radial growth behavior of tested PSB.

PSB strains/Fungal strains	Commercial control	WJEF15	WJEF26	WJEF38	WJEF39	WJEF41	WJEF45	WJEF46	WJEF51	WJEF56	WJEF59	WJEF61	WJEF63
*A. niger*	21.31	–	23.21	–	–	–	–	–	21.42	–	49.15	–	–
*F. culmorum*	–	20.44	–	18.97	–	–	18	24	18.36	–	23.51	22.44	–
*F. oxysporum*	18.64	–	21.15	–	22.92	–	–	16.67	–	–	32.76	–	–
*P. digitatum*	26.92	–	–	–	–	33.89	–	–	20.33	–	–	–	–
*P. notatum*	25.53	–	16.03	–	–	–	–	–	18	–	–	–	–
*R. stolonifer*	–	23.07	21.15	17.31	26.93	30.77	25	–	–	36.54	32.92	19.23	21.42

## Discussion

The present study focused on the effect of several tolerant PSBs on different abiotic stresses to promote P solubilization from RP and TCP as the only P source in the context of biological fertilization and sustainable agricultural strategy. Increasing soil P concentrations *via* inorganic and organic soil P mobilization pools involve changes in pH and the release of P-mobilizing compounds such as carboxylates and phosphatases, which are exuded by the roots themselves or by micro-organisms in the rhizosphere. However, the selection of an efficient PSB is crucial, as it practically increases P in the plant rhizosphere (Aliyat et al., 2020). Phosphate solubilization capacity is considered a key factor in isolating highly efficient PSBs from agricultural soils (Aliyat et al., 2020). Among 134 PSB strains from different leguminous plants’ rhizosphere in the Fez-Meknes region, only 12 (12) PSBs show phenotypic stability of P solubilization after five successive subcultures in the NBRIP-RP plates.

The solubility index (Figure 2) indicates a PSI of TCP in agreement with previous studies conducted on different plants’ rhizosphere, where the PSBs solubility index ranges from 2 to 5.1 (Sadik et al., 2013; Rfaki et al., 2014; Manzoor et al., 2017). The PSB isolated strains used in this work were able to increase P availability in NBRIP liquid medium containing RP or TCP as a source of P, also a combination of RP * TCP. Our results support the idea that PSBs are characterized by their ability to easily and efficiently solubilize inorganic P (Elkoca et al., 2008; Anand et al., 2016; Adnan et al., 2019). This is confirmed with the use of *Bacillus subtilis* DSM 10, which improved the P nutrition of common beans in hydroponic experiments when plants were inoculated with *Rhizobium tropici* and grown with one of the two P sources (Pi or phytate) and co-inoculated with *B. subtilis* (Maougal et al., 2014). Our results show that soluble P concentration in the medium containing RP was between 16.11 mg.l^–1^ and 25.16 mg.l^–1^, while the medium containing TCP was between 75.61 mg.l^–1^ and 147.62 mg.l^–1^ (Figure 3). These results are in agreement with other studies done by Zaidi and El Naqa (2010) and Anand et al. (2016), who report that PSBs can solubilize insoluble inorganic P compounds, such as TCP, dicalcium P (DCP), hydroxyapatite, and RP. Phosphate solubilization is accompanied by a pH medium decrease when compared to control, both in media containing RP and TCP. Furthermore, we note that as pH values decrease, the P concentration increases. This is noted by Batool and Iqbal (2019), who indicate a strong correlation between P solubilization and pH decrease. Changes in pH value in the rhizosphere, caused by roots or microorganisms, can influence the surface charges on metal oxide particles and thus Pi availability (Hinsinger et al., 2009), ultimately leading to Pi release that is available to plants. P mobilization in the rhizosphere is largely due to pH changes induced by roots and/or associated microorganisms and to carboxylate exudation (Hinsinger et al., 2011). Soil pH is critical in determining the availability of inorganic forms of P (Hinsinger et al., 2009; Adnan et al., 2018), and it can also influence the fate of organic P through its impact on enzymatic activities. In neutral to alkaline soils, Pi occurs largely in various forms of Ca phosphate (mainly apatites) whereas, in acidic and deeply weathered soils, such as those abundant in the tropics, much of the soil Pi occurs as bound to and/or occluded in Fe and Al oxy (hydr) oxides (Jones and Oburger, 2011). As the positive charges on these minerals become increasingly positive with decreasing pH, greater amounts of P can be adsorbed at low rather than high pH. In addition, Ca-phosphates solubility is strongly increased with decreasing pH. Devau et al. (2011) showed that both increasing and decreasing pH could lead to an increase in soil P availability, which was consistent with the findings of previous studies, as reviewed by Hinsinger et al. (2009). Similarly, Alori et al. (2017) observed a negative correlation between the amount of P solubilized by *B. cepacia* (SCAUK0330) and decreasing pH, leading to increased solubilization of P. As proposed by Batool and Iqbal (2019), the decrease in pH can be explained by organic acid production and H^+^ secretion. Several studies support this argument and indicate that the main mechanism of mineral P solubilization is lowering soil pH by microbial organic acid production (Rodríguez and Fraga, 1999; Khan et al., 2010; Sharma et al., 2013; Anand et al., 2016). The excretion of organic acids that promote Pi solubilization is a trait found in many P-solubilizing microorganisms. Rodríguez et al. (2007) listed several bacterial genes involved in gluconic acid oxidation, which is a major mechanism of mineral P solubilization in Gram-negative bacteria (Chauhan H. et al., 2015).

Experimental design results of P solubilization and pH medium are highly significant and show that pH medium and P[C] is maximal when RP and TCP are at the highest level (2.5 and 2.5%) (Tables 6, 7). However, Bashan et al. (2013) pointed out that the most commonly used source of insoluble Pi, tricalcium phosphate (TCP), is unreliable and insufficient; And also suggested that a combination of two or three metal-P compounds is used together or in tandem, depending on the type of soil and final use of screened bacteria. This would result in a significant reduction in potential PSBs and would also maximize the chances of selecting the most efficient strains capable of contributing to plant P nutrition. Our experiments show an increase in P solubilization accompanied by a decrease in pH, increasing as RP and TCP concentration increase (Figures 5, 6). Similarly, Yadav et al. (2017) use organic compost with RP at different concentrations to optimize P solubilization by PSBs. They found that the 75:25 ratio of RP and compost showed maximum soluble P accompanied by higher plant growth when compared to plants fertilized with normal compost and rock phosphate. Our result can explain the Pi solubilization process by micro-organisms due to carboxylates and organic acids production. Concerning the free extracellular phosphatase enzymes, although it is difficult to differentiate their origin (Burns et al., 2013), several examples show that microbial enzymes are predominant in soil (Gao et al., 2019), and these present a higher efficiency for Pi release (Adnan et al., 2019). Different types of phosphatases can be found, including phosphomonoesterases, which have been most studied, especially for P-solubilizing microorganisms (Jones and Oburger, 2011).

On the other hand, as discussed by Khan et al. (2010), although there is a clear potential for the development of inoculants with PSBs, their widespread application remains limited by a poor understanding of microbial ecology, dynamics population in soil, and inconsistent performance in a range of environmental factors. However, although PSBs are present in the majority of soils, their performance is strongly influenced by environmental factors such as soil type, temperature, salinity, cation exchange capacity, pH, organic matter, and availability of substances in soils (Rfaki et al., 2018; Masdariah et al., 2019). Similarly, Namli et al. (2017) stated that resistance to extreme environmental conditions is an important characteristic that allows inoculant bacteria to survive and maintain optimal population numbers throughout a specific crop life cycle. Resistance to intrinsic and extrinsic stresses is an important factor for the proliferation, development, and survival of microorganisms in rhizospheric soils. In our study, to have a more appropriate approach for competent strains selection of PSB that could increase the chances of successful field inoculation and significantly improve plants’ P status, all selected strains showed good tolerance to various environmental stresses such as increased temperature from 37 to 55°C, salinity ranging from 0.2 to 2 M, pH between 3 and 13 (Table 9), and drought variation of PEG-600 from 10 to 30% (Figure 8). Therefore, these microorganisms that survive under drastic conditions could be used in different cultural practices to improve agricultural production. Moreover, isolated PSBs showed a strong ability to inhibit different fungal species (Table 10). These results were confirmed by the findings of Muleta et al. (2009), who reported that several *Pseudomonas* strains which could inhibit phytopathogenic strain *Fusarium oxysporum* possess the ability to produce HCN, siderophores, and antibiotics.

Furthermore, Gram staining indicates a group of Gram-negative and Gram-positive strains, with an advantage for Gram-negatives (Table 8). Besides, previous observations have shown that several plants’ rhizosphere selects more Gram-negative than Gram-positive rhizobacteria (Muleta et al., 2009). The cell motility test indicates that all selected strains were motile (Table 8). Moreover, bacterial motility is considered to be an essential element for successful root colonization and P transfer in soil (Shahid et al., 2015). In addition, it has been reported that motile bacteria have better access to root exudates in the rhizospheric zone through chemotaxis (Shahid et al., 2015; Manzoor et al., 2017). Motility can potentially enhance the rhizoplane competence in terms of movement, both from bulk soil to root and along roots (Dutta and Podile, 2010), leading to heterogeneous colonization of host plant roots by the PGPR. After root surface colonization, PGPR can stimulate plant growth through several mechanisms impacting plant nutrition and, in some cases, also improving its resistance to pathogens (Pérez-Montaño et al., 2014).

However, PSB biotechnology offers an excellent opportunity to develop durable biofertilizers to be used as supplements and/or alternatives to chemical fertilizers to overcome agricultural challenges imposed by an ever-increasing food demand (Sharma et al., 2013). Moreover, with readily available carbon resources, free-living soil bacteria can rapidly mineralize P from soil organic matter and incorporate it into their biomass. Microbial P thus represents not only an important P sink in soil and terrestrial ecosystems but also a possible source of available P for plants (Xu et al., 2013). In addition to chemical fertilizers, PSBs application as biofertilizers could also promote plant growth with large amounts of soluble P (Pi, polyphosphates, and organic P) released from lysed bacterial cells following rewetting of dry soils or during freeze-thaw cycles at the beginning of the growing season. For example, Turner et al. (2003) showed that the potential contribution of bacterial cell lysis to water-extractable phosphorus following soil drying varied between 88 and 95% in two Australian pasture soils. In general, the selection of a competent strain of PSBs as an inoculant and their qualification as a biofertilizer should not only be based on laboratory and greenhouse experiments but also on field experiments.

## Conclusion

The main objective of this study was to assess several PSB strains’ ability to enhance solubilization activity from RP, TCP, and their combination. As a problem, inorganic P fixation into insoluble complexes renders these compounds inaccessible for plant absorption. The use of biofertilizers such as microorganisms can help in this regard by sustainably enhancing plant growth. Based on the mentioned results, we find that the isolated PSB strains have an antifungal effect of up to 49.15% inhibition as well as a high capacity to resist intrinsic and extrinsic stress. In addition, we note that P solubilization significantly increased by 20.58% when RP and TCP percentages in combination are at the max level. By analyzing all the different orientations of this study, we recommended the use of PSBs strains with high potential as microbial fertilizers in the agronomic field. To exploit the full potential of these telluric bacteria, more studies are need to be investigated on the genetic mechanisms level and microbial-mineral interfaces. Further innovative research should focus on the application concepts of microbial biotechnology in agriculture to identify more PSBs for their usage in a consortium to have competent microbial inoculants in sustainable cultures’ production systems under various conditions.

## Data availability statement

The original contributions presented in this study are included in the article/supplementary material, further inquiries can be directed to the corresponding author/s.

## Author contributions

All authors listed have made a substantial, direct, and intellectual contribution to the work, and approved it for publication.
